# Markers for discriminating *Campylobacter concisus* genomospecies using MALDI-TOF analysis

**DOI:** 10.1016/j.crmicr.2020.100019

**Published:** 2020-12-16

**Authors:** Stephen L.W. On, Junwen Zhang, Angela J. Cornelius, Trevor P. Anderson

**Affiliations:** a Wine, Food and Molecular Biosciences, Lincoln University, PO Box 85084, Lincoln, New Zealand; b Institute of Environmental Science, Christchurch Science Centre, Christchurch, New Zealand; c Canterbury Health Laboratories, Hagley Avenue, Addington, Christchurch, New Zealand

## Abstract

Strains identified as *Campylobacter concisus* may belong to one of at least two biochemically indistinguishable, but genomically distinct, groups referred to as “genomospecies” that may differ in their pathogenic and zoonotic potential. Reliable, affordable and available identification methods are required to improve understanding of their significance in human illness. We examined the potential for MALDI-TOF MS, increasingly used in routine laboratories, for this task. Nineteen well-characterised strains were examined using a widely used MALDI-TOF MS commercial system, however only one strain confidently identified using their database. Data mining of the spectra obtained revealed a number of markers that could be used to help discriminate these genomospecies. We conclude that careful application of MALDI-TOF analysis could be useful to determine the role and significance of diverse *C. concisus* genomospecies in human disease.

## Highlights

• Efficacy of commercial MALDI-TOF system for identifying *C. concisus* genomospecies evaluated.
• Use of cluster analysis of MALDI-TOF profiles to discriminate *C. concisus* genomospecies explored.
• MALDI-TOF-based markers helpful for identification of *C. concisus* genomospecies identified.

## 1 Introduction

Since its initial isolation and description from the oral cavity of humans with periodontal disease (Tanner et al., 1981), *Campylobacter concisus* has been associated with a range of gastrointestinal ailments, including acute and chronic diarrhoea (Aabenhus et al., 2002; Cornelius et al., 2012; Lastovica, 2009; Tanner et al., 1981; Underwood et al., 2016; Vandamme et al., 1989), inflammatory bowel syndrome (Kirk et al., 2018; Zhang et al., 2010, 2014), Crohn's disease (Guslandi et al., 2009; Liu et al., 2018), and microscopic (Yde Aagaard et al., 2020) and ulcerative (Liu et al., 2020) colitis . Its presence in domestic pet dogs infers potential for zoonotic transmission (Chaban et al., 2010). However, *C. concisus* may also be found in humans with good oral health (Ismail et al., 2012) and in faecal samples from individuals in which gastrointestinal disturbances have not been detected or diagnosed (Cornelius et al., 2012; Van Etterijck et al., 1996).

These apparent conflicts as to the pathogenic potential of the organism has been proposed by some to be explained by its extensive genetic diversity, which has been demonstrated in various studies (Aabenhus et al., 2005; Kirk et al., 2018; Liu et al., 2020; Matsheka et al., 2002). Critically, strains identified as *C. concisus* may in fact belong to closely related, yet genetically distinct taxa referred to as “genomospecies”, of which two appear predominant (Aabenhus et al., 2005; Liu et al., 2020; Mahendran et al., 2015; Vandamme et al., 1989). Multi-Locus Sequence Typing (MLST) generally supports a phylogenetic distinction between these two groups, although the separation is not perfect (Miller et al., 2012). The *C. concisus g*enomospecies could be proposed as nomenclaturally distinct species if a clearly defined, readily determined phenotypic marker were identified, however at this time no such trait has been found. This hinders the accurate attribution of the major *C. concisus* genomospecies in healthy and diseased individuals, and in domestic pets, to improve our understanding of the public health impact of these bacteria. Given the frequency that *C. concisus* has been found in human faeces (Cornelius et al., 2012; Lastovica, 2009; Nielsen et al., 2013), this is an important question to resolve.

Microbial identification in routine laboratories has been transformed in many countries with the implementation of commercial platforms to undertake Matrix-Associated Laser Desorption/Ionisation – Time-Of-Flight Mass Spectroscopic (MALDI-TOF MS) analysis (van Belkum et al., 2015). This method involves the breakdown of cells by laser energy into molecules that are separated and detected on the basis of their differing mass and electrical charge. The resulting spectrum can then be compared to databases containing similar spectra derived from those of known organisms and an identification attained when a threshold similarity level is reached. This process is automated in commercial systems, but is reliant upon the relevant taxon being present in the database.

This paper examines the performance of a commercial MALDI-TOF MS identification system for strains assigned to each of the two major *C. concisus* genomospecies, and explores the potential for enhanced discrimination using this method.

## 2 Materials and methods

### 2.1 Strains examined

Strains examined and their sources are listed in Table 1. Genomospecies attribution had been determined previously by DNA-DNA hybridisation and/or Amplified Fragment Length Polymorphism (AFLP) analysis (On et al., 2013; Vandamme et al., 1989).Where whole genome sequences and MLS types are available, these details are also given. According to AFLP profiling, all strains were distinct (On et al., 2013).

**Table 1:** Genomospecies designations and sources of *Campylobacter concisus* strains examined, and summary of their identification results with the commercial (Bruker) database. Genbank accession numbers for genome sequences and Multi-Locus Sequence (MLS) Types (Miller et al., 2012) where available are also listed. All newly determined MLS types were unique.

Genomospecies designation	Strain no.	Source	Whole-genome accession no.	MLS type	Bruker database classification (no. of replicates/no. samples)
1	CCUG 13144^T^	Adult, gingivitis, USA	CP01254	77	CI (24/24)
1	L24.99	Faecal, fever			PGI (2/2)
1	L28.99	Bloody diarrhoea	NDYO00000000	New	PI (1/2); PGI (1/2)
1	L61.99	Dysentery, 12 months, *Shigella dysenteriae* co-isolated	NEFM00000000	New	PGI (1/1)*
1	L64.99	Bloody diarrhoea	NDYP00000000	New	PI (22/24); PGI (2/24)
1	L115.99	Faecal, aplastic anaemia		29	PI (2/2)
1	L220.96	Bloody diarrhoea	NDYS00000000	25	PGI (2/2)
1	L389.96	Chronic diarrhoea			PI (1/2); PGI (1/2)
2	CCUG 19,995	Faeces, adult, recurrent fever and exanthema, Sweden	NDYN00000000	New	PI (15/24); PGI (9/24)
2	L104.93	Loose stools			PGI (2/2)
2	L113.99	Prolonged diarrhoea, *Cryptosporidium* spp. co-isolated			PI (13/24); PGI (11/24)
2	L127.99	Chronic diarrhoea	NDYQ00000000	39	PGI (2/2)
2	L131.99	Dysentery, *S. flexneri* co-isolated		26	PGI (2/2)
2	L135.99	Faecal, maladsorption, *S.dysenteriae* co-isolated			NI (2/2)
2	L140.99	Bloody diarrhoea			PI (1/2); PGI (1/2)
2	L275.95	Rectal prolapse and diarrhoea,*Trichuris* spp. co-isolated			PI (1/2); PGI (1/2)
2	L312.98	Chronic diarrhoea			PGI (1/2); NI (1/2)
2	L316.98	Dysentery, *S. flexneri* co-isolated			PI (1/2); PGI (1/2)
2	L377.96	Bloody diarrhoea		21	PI (1/2); PGI (1/2)

T=Type strain. CCUG=Culture collection of the University of Gothenberg, Sweden. *L*=from the collection of Prof. A. Lastovica, Cape Town. S. Africa. ^⁎^=one sample failed to yield peaks. Bruker identification codes (scores): CI, Correct species identification (2.3 −3.0). PI, Probable Species/secure genus (2.0–2.299). PGI, Probable Genus Identification (1.7–1.99). NI, Not reliably Identified (0–1.699).

### 2.2 MALDI-TOF MS analysis and routine identification of strains

Strains were cultured for 3 days under microaerobic conditions (80% N_2_, 10% CO_2_, 3% O_2_, 7% H_2_) in a dedicated workstation (Don Whitley Scientific, Bingley, UK) on 5% blood agar. MALDI-TOF MS profiling was performed using a Flex Biotyper instrument (Bruker Diagnostics, Karlsruhe, Germany). Discrete samples of bacterial growth were smeared onto the steel analysis plate and 1 µl of 70% formic acid added before addition of the matrix solution; samples were air dried before analysis, as described previously (Werno et al., 2012). For four strains, 24 samples of bacterial growth were examined (the number recommended for determining reference samples), and for the remaining 15 strains, duplicate samples were examined. The resulting spectra were then compared to existing data in a proprietary database (v.6903; Bruker Diagnostics) using manufacturers recommended guidelines, as described previously (Ge et al., 2017). The proprietary software evaluates the degree of resemblance of profiles, and outlines the best identification to its database entries (*n* = 6903 as of this study), as follows. Highly probable species identification (2.3-3.0); secure genus identification, probable species identifications (2.0-2.299); probable genus identification (1.7-1.999); or not reliable identification (0-1.699). Samples were compared with the Bruker database on 7th October 2020.

### 2.3 Cluster analysis and biomarker identification

Strain MALDI-TOF MS spectra were exported in a textfile format, and assimilated into the software BioNumerics 7.6 (Applied Maths, Kortrijk, Belgium) for analysis. Cluster analysis was performed using the Peak based-Dice coefficient using the parameters of minimum height 0%, peak matching of constant tolerance 1, linear tolerance 500 ppm, shift factor 1, and UPGMA (unweighted-pair group method with arithmetic mean) algorithm.

The potential biomarkers were identified using the matrix mining tool according to the BioNumerics Tutorial “Peak matching and follow up analysis of spectra”. The peak matching was performed using default settings (constant tolerance 1.9, linear tolerance 550 ppm, peak detection rate 10). All peak classes with a *p*-value < 0.05 were initially selected and further considered as the potential biomarker, combined with visual observation.

## 3 Results

### 3.1 Identification of *C. concisus* strains using the proprietary bruker database

The range of identification scores for each of the MALDI-TOF sample replicates for the strains examined is summarised in Table 1. Only the type strain of *C. concisus* was consistently confidently (24/24 replicate samples with scores >2.3; 40.6% of all genomospecies 1 samples tested) identified to this species, and this was the only strain we tested that is present in the Bruker database. Of the remaining *C. concisus* genomospecies 1 strains, 45.7% of samples were considered probable species identifications and 13.5% probable genus identifications, in each case with *C. concisus* named as the most likely species. Genomospecies 2 strains yielded less definitive results, with 55.1% samples considered probable to species level, and 39.6% considered probable to genus level, again with *C. concisus* named as the most likely species. However, 5.1% of samples were not identified to any defined species (Table 1).

### 3.2 Cluster analysis of MALDI-TOF spectra of *C. concisus* genomospecies

**Fig. 1:**
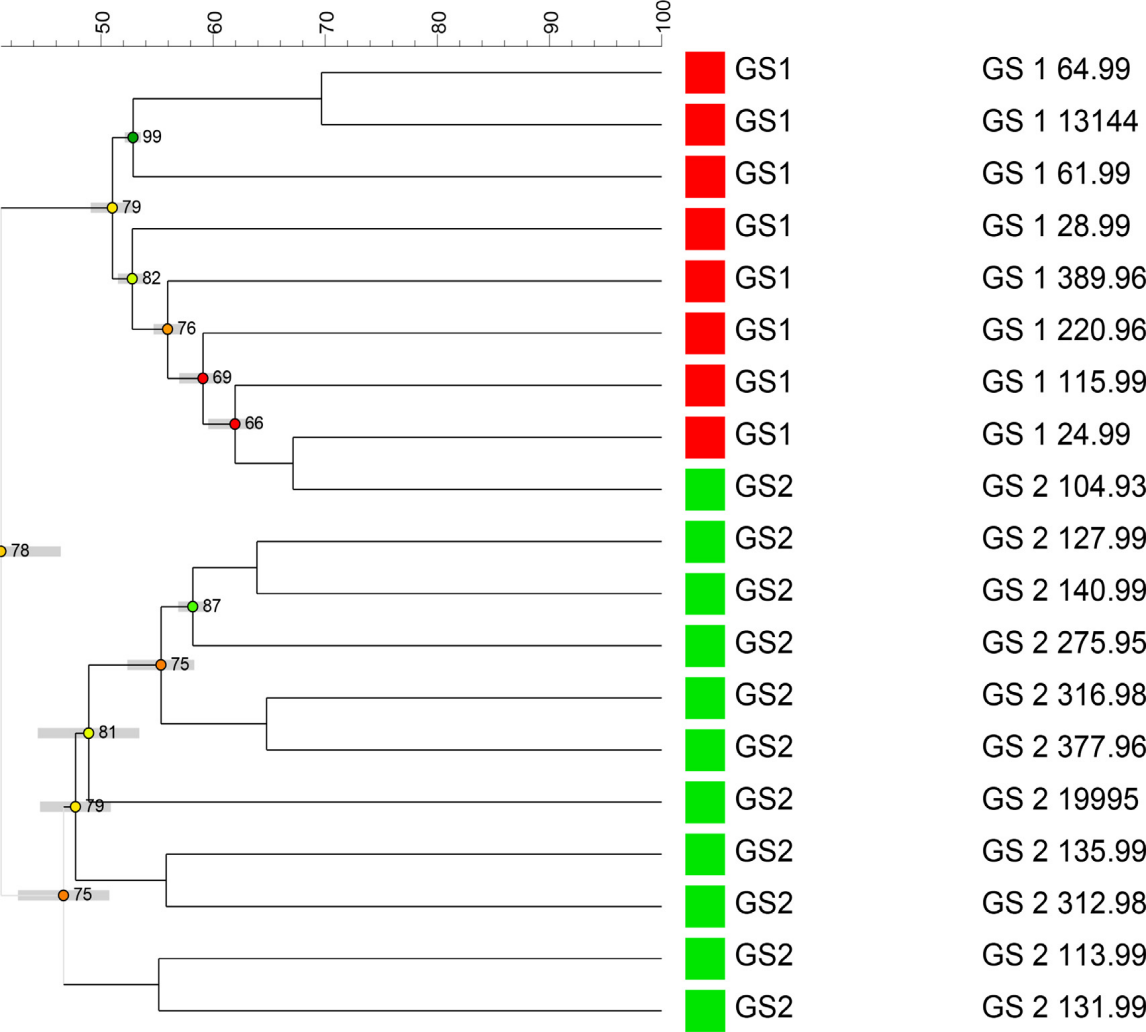
Cluster analysis of the MALDI-TOF MS spectra determined for the 19 *C. concisus* genomospecies (GS) strains examined.The scale bar along the top of the dendrogram represents the per cent similarity calculated between the strains by the Dice coefficient and UPGMA clustering. Figures displayed on the nodes of clusters containing ≥3 strains represent cophenetic correlation values that evaluate the congruence between the similarity matrix and the dendrogram.

Two major clusters were formed at the 44% similarity level (Fig. 1). The first contained each of the eight Genomospecies 1 strains, plus one GS2 strain (L104.93). The second comprised GS2 strains only. The relatively low level of similarity exhibited among the strains is indicative of substantive diversity among the spectra.

### 3.3 MALDI-TOF MS markers of *C. concisus* genomospecies

Table 2 lists marker peaks identified by the BioNumerics software, and confirmed by careful scrutiny of these data, that provided discrimination between the two genomospecies. Minor differences in the m/z values displayed were not considered by the software to be sufficient to distinguish them and also could not be differentiated when displayed in the cluster analysis (data not shown). Fourteen markers were considered useful for differentiation of the subspecies, although only one (m/z 7634.66) provided clear discrimination, being found only among GS1 strains.

**Table 2:** Discriminatory markers of *C. concisus* genomospecies based on MALDI-TOF MS analysis.

		Marker no./*M/Z* peak position												
	1	2	3	4	5	6	7	8	9	10	11	12	13	14
**Genomospecies/ Strain no.**	**3150.21**	**3459.84**	**3465.59**	**3666.46**	**3982.42**	**6915.61**	**6927.94**	**7429.94**	**7634.66**	**8048.76**	**8996.27**	**9463.97**	**10,377.06**	**10,456.04**
**GS 1 L64.99**	3151.39	3457.00	–	–	3981.47	6913.73	–	–	7633.35	–	8990.64	9462.75	–	10,452.26
**GS 1 CCUG 13,144**	–	3457.70	–	–	–	6915.00	–	–	7631.70	–	8990.65	9465.00	–	10,452.80
**GS 1 L61.99**	–	3458.10	–	–	3982.00	6914.20	–	–	7635.00	–	–	9462.90	–	10,452.80
**GS 1 L28.99**	–	3457.60	–	–	–	6914.25	–	–	7633.60	–	–	9463.05	–	–
**GS 1 L389.96**	–	3459.25	–	–	3982.75	6915.95	–	–	7635.60	–	8992.50	–	–	10,462.40
**GS 1 L220.96**	–	3457.75	–	–	3981.65	6913.70	–	–	7634.25	–	8994.05	9460.45	–	10,452.80
**GS 1 L115.99**	–	3459.15	–	–	3983.60	6916.75	–	–	7638.45	–	9001.00	9465.85	–	10,457.60
**GS 1 L24.99**	–	3460.10	–	–	3982.35	6916.35	–	–	7632.95	–	8998.15	–	–	–
GS 2 L104.93	–	3461.55	–	3667.85	–	6918.70	–	7432.00	–	8052.55	–	9464.60	–	–
**GS 2 L127.99**	3152.10	–	3465.55	3666.40	–	–	6928.30	7430.55	–	–	–	–	10,377.20	–
**GS 2 L140.99**	3150.00	–	3465.05	3666.65	–	–	6927.00	7431.10	–	8048.10	–	–	10,374.75	–
**GS 2 L275.95**	3146.25	–	3465.40	3666.00	–	–	6927.50	7429.85	–	8049.25	–	–	10,376.80	10,462.40
**GS 2 L316.98**	–	–	3465.30	–	–	–	6928.35	7430.70	–	8051.60	–	–	10,378.25	–
**GS 2 L377.96**	3150.70	–	3465.40	3666.70	–	–	6928.85	7431.40	–	8051.40	–	–	10,377.55	–
**GS 2 CCUG 19,995**	–	–	3462.85	–	–	–	6924.60	7427.00	–	8045.90	–	–	–	–
**GS 2 L135.99**	3149.80	–	3465.25	3666.40	–	–	6927.35	–	–	–	–	–	–	–
**GS 2 L312.98**	–	–	3465.90	3666.35	–	–	6926.85	–	–	–	–	–	–	–
**GS 2 L113.99**	3152.30	–	3465.63	3666.93	–	–	6929.00	7432.65	–	–	–	–	–	–
**GS 2 L131.99**	3151.85	–	3466.20	3667.30	–	–	6932.40	7433.00	–	–	–	–	–	–

## 4 Discussion

The genotypic variability of the *C. concisus* genomospecies has been well documented (Aabenhus et al., 2005; Kirk et al., 2018; Liu et al., 2020; Matsheka et al., 2002) and likely underpins the substantive phenotypic diversity observed in this study, and indeed in others where whole-cell protein profiling has been used (Vandamme et al., 1989). The variation demonstrated illustrates the difficulties that have been encountered for many years in identifying a single discriminating marker for these two taxa; the absence of a simple such differential feature is the fundamental reason that these taxa have not been formally described as distinct species, as clearly indicated by their whole-genomic relatedness (Vandamme et al., 1989). In this respect, it is encouraging to note that we have determined the presence of a single marker that is present only among GS1 isolates. Further investigation and characterization of this trait may provide an important key towards the development of a simple test that can then be applied for easy genomospecies discrimination and subsequent description of novel species in accordance with minimal taxonomic standards (On et al., 2017).

As of 7th October 2020, the Bruker identification database contained 14 strains of *C. concisus,* including the type strain examined in our study. The genomospecies designations of the other strains is not known and clearly do not represent the full diversity of *C. concisus* phenotypes, since 18 of our 19 isolates did not achieve convincing identification scores. Such performance can easily be improved by incorporating our data into the proprietary database and the manufacturer has a standard protocol by which this can be achieved.

The role of *C. concisus* genomospecies in gastrointestinal disease and potential zoonotic infection has long been difficult to resolve given the problems in routinely identifying them. It is hoped this study provides some insights into the use of an increasingly available tool, MALDI-TOF MS, for achieving this aim.

## Declaration of Competing Interest

The authors declare that they have no known competing financial interests or personal relationships that could have appeared to influence the work reported in this paper.
